# Teacher Violence and Student Wellbeing in Rural Sierra Leone: Longitudinal Dynamics Across Primary Schooling

**DOI:** 10.3390/bs14111106

**Published:** 2024-11-18

**Authors:** Giulio D’Urso, Jennifer Symonds, Seaneen Sloan, Daniel Capistrano, Elena Samonova, Dympna Devine, Ciaran Sugrue

**Affiliations:** 1 Department of Law, Economics, and Human Sciences, Mediterranean University of Reggio Calabria, 89124 Reggio Calabria, Italy; 2 Faculty of Education and Society, University College London, London WC1E 6BT, UK; j.symonds@ucl.ac.uk; 3 School of Education, University College Dublin, D04W6F6 Dublin, Ireland; seaneen.sloan@ucd.ie (S.S.); dympna.devine@ucd.ie (D.D.); ciaran.sugrue@ucd.ie (C.S.); 4 School of Sociology, University College Dublin, D04W6F6 Dublin, Ireland; daniel.capistrano@ucd.ie; 5 Institute of Geography, University of Bremen, 28359 Bremen, Germany; elena.samonova@ucd.ie

**Keywords:** violence, students, teachers, wellbeing, Sierra Leone

## Abstract

This study explored the longitudinal dynamics of teacher violence and student wellbeing in rural Sierra Leone, West Africa. The participants, totaling 3170 children with an age range of 5 years to 11 years, were cluster-sampled from a large geographic area to ensure gender balance and representation from diverse linguistic backgrounds and religious affiliations. They were drawn from the Safe Learning Study, which spanned over 5 years and involved 100 schools in rural Sierra Leone. Data collection took place in four waves from November 2018 to May 2021. Participants completed self-report questionnaires pertaining to psychological wellbeing and experiences of violence from teachers. The study employed a random intercept cross-lagged panel model (RICLPM) to examine the relationship between violence and mental health across waves. Across children, a relationship between teacher violence and student wellbeing was observed over time. However, for individual children, higher wellbeing predicted lower instances of violence, and vice versa, although to a weak extent. These findings highlight the complex interplay between violence and wellbeing within the cultural sample. These insights contribute to a deeper understanding of the social dynamics surrounding violence and wellbeing, informing targeted interventions and policy initiatives aimed at creating safer and healthier environments for at-risk populations.

## 1. Introduction

### 1.1. Definition and Spectrum of Violence

Violence is defined as an extreme form of aggression, whether physical or verbal, intended to inflict severe physical harm, such as serious injury or death [1,2]. Violent behaviors can be understood as existing along a continuum of severity. On this spectrum, less severe acts, such as pushing or shouting, fall on the lower end, while more extreme acts, such as physical assault or homicide, occupy the higher end. Importantly, while all violent acts are forms of aggression, not all acts of aggression qualify as violence. For example, pushing another person in a non-lethal confrontation is an aggressive act, but it is not violent by definition. In a socio-cultural context, a child pushing another child to claim a toy would typically be classified as aggression but not as violence, as the intent and severity of harm are considerably lower [3]. This distinction is critical for understanding the different levels of aggressive behavior and how they manifest in various situations. The literature also includes forms of child maltreatment and sexual abuse in the category of violence against children, generally occurring in family and school contexts [4]. The World Health Organization (WHO) [5] defines violence as the intentional use of (physical) force or power, threatened or actual, against oneself, another person, or a group or community, which either results in or has a high likelihood of resulting in injury, death, psychological harm, maldevelopment, or deprivation. To summarize, violence encompasses any form of mistreatment that occurs along a continuum of severity and can manifest in various forms. It may be physical, verbal, or a combination of both. This means that violence is not limited to just physical harm but can also include verbal abuse, such as insults or threats, which can be equally damaging. The different forms of violence may overlap, and their impact on the individual can vary in intensity depending on the nature and frequency of the abuse.

### 1.2. Violence in Sierra Leonean Schools

Violence and abuse are prevalent in Sierra Leonean schools and can include physical, psychological, and sexual forms of harm. Corporal punishment is extensively practiced, with most boys and girls experiencing caning or whipping, which they often view as a routine aspect of their school experience [6]. The literature also indicates that in numerous poverty-stricken countries, such as Sierra Leone, violence and corporal punishment (e.g., beatings) are culturally deemed acceptable methods for enforcing the rule of law, as determined within the cultural context [7]. There, physically punishing children is not necessarily seen in a negative light [8]. For instance, in several parts of Africa, adults describe physical punishment as a way of showing love for children and argue that not disciplining children when necessary is neglectful [9]. Nevertheless, the Sierra Leone Child Rights Act (2007) emphasizes that social systems must protect children and safeguard them from all forms of violence and abuse. The implementation of various projects and increased education on children’s rights have highlighted that corporal punishment and child labor are violations of children’s rights [10].

The literature highlights how children in Sierra Leone are at risk of violence and various forms of abuse perpetrated by significant figures in their lives [11]. The literature emphasizes how children in Sierra Leone are particularly vulnerable to various forms of violence and abuse, often perpetrated by significant figures in their lives, such as family members and teachers. According to Amowitz et al. [11], many children are victims of physical, emotional, and sexual abuse, which have devastating consequences on their psychological and physical development. This situation is further exacerbated by the broader socio-economic context of the country, where a lack of social support and, in many cases, the indirect or direct tolerance of violence, perpetuates these abuses.

In Sierra Leone, indeed, the cultural and religious context plays a crucial role in shaping societal attitudes toward discipline and child-rearing practices. A significant portion of the population adheres to Islam, where corporal punishment may be viewed as an acceptable or even necessary form of discipline, in line with traditional interpretations of religious teachings. However, while these practices are often rooted in the intention to instill respect and moral values, the potential harmful effects on children’s psychological and emotional wellbeing are frequently underestimated. Research shows that corporal punishment, regardless of its perceived disciplinary benefits, can lead to long-term emotional trauma, fear, and hindered cognitive development in children [12]. It is essential to distinguish between religious beliefs that emphasize moral education and practices that, though culturally accepted, may inadvertently perpetuate cycles of violence and emotional harm.

### 1.3. Teacher Violence and Its Consequences

Studies conducted across West Africa have also found that many children are exposed to high levels of violence, which is closely linked to wellbeing and general mental health [13,14]. The teaching and learning environment in low-income countries is currently fraught with multiple risk factors, partially attributable to teachers’ use of violence [15]. Teacher violence is considered a form of school violence, involving any physical, psychological, or verbal aggression from teachers to students, which is still prevalent, especially in countries such as Sierra Leone [16]. Corporal punishment at school is still very common in different parts of the world [17,18]. Indeed, a study conducted by Mncube and Netshitangani [19] in Africa suggested that many teachers are verbally, physically, and psychologically violent toward pupils. A study conducted by Meyer and colleagues [20] suggested that the lack of a positive school environment and children’s exposure to community violence—which in turn is linked to negative mental health outcomes—are associated with teachers’ use of violence in schools in low-income countries. The literature highlights how teacher physical violence can affect the wellbeing of children, who may become extremely vulnerable [13,21,22] Contrary to expectations, this suggests that those with higher academic attainment would report a higher sense of wellbeing. However, the literature shows conflicting results. For example, a study by Noreen et al. [21] highlighted both a positive association between corporal punishment and academic performance and, at the same time, a negative association between corporal punishment and psychological wellbeing. Therefore, while corporal punishment might correlate with improved academic performance, its detrimental effects on psychological health cannot be overlooked. Physical violence and punishment perpetrated over time by significant figures in children’s lives can be risk factors related to the onset of internalizing or externalizing psychopathologies [23,24,25]. However, teachers often justify using violence as part of disciplinary procedures, believing it helps maintain authority, command respect, and control student behavior [26,27].

The literature highlights how the severity and the frequency of the violence may be considered a traumatic event that can make children vulnerable in the development of socio-emotional skills [28,29,30]. Bifulco and colleagues [31] suggested that experiences of traumatic events may impact the wellbeing of children, as they harm the intimate and deep spheres of the self during the early stages of development. The frustration derived from such experiences may result in the psychic structure of children being compromised, and consequently, they may struggle to internalize positive behavioral models such as empathy, conflict resolution, and cooperation. In the cycle of violence, the greatest risk is not only to the wellbeing and mental health of the child but also to the development of conditions in which the child considers violence as a possible solution to obtain respect and control of a situation [32]. However, there is a lack of studies linking violence inflicted by teachers and wellbeing, as this is an under-researched area, although more prevalent in middle-income countries and in high-risk contexts of violence [33]. A recent meta-analysis highlighted the significant psychological effects of experienced violence, including internalizing behaviors at the individual level [34]. This underscores the necessity of considering, in accordance with the ecological model, the impact of violence at both the individual and school levels. At the individual level, traumatic experiences can profoundly affect children’s internal emotional states, leading to a general sense of wellbeing that is often overlooked, especially in contexts like Sierra Leone [35] where strict disciplinary practices are normative. In such environments, children may feel compelled to internalize their distress, fearing that expressing their feelings could lead to further punishment or social stigmatization. At the school level, the consequences of teacher violence can manifest in various outcomes that are not yet clear. On one hand, such violence can significantly diminish students’ overall wellbeing; on the other hand, in a context where violence is perceived as a normative practice, the impact may not be as pronounced or may even be normalized by students, leading to a desensitization to violence. This duality presents challenges in assessing the true effects of violence in educational settings in Sierra Leone, where cultural attitudes toward discipline can complicate the recognition of violence as a detrimental factor in student wellbeing.

## 2. The Current Study

Based on this theoretical framework, which reveals some controversy regarding the effects of violence in Sierra Leone, and considering that there are no studies that have examined the reciprocal association between teacher violence and children’s wellbeing at both the individual and school levels, this study aims to fill this gap. Specifically, it seeks to investigate the extent to which teacher-inflicted violence impacts children’s wellbeing within the school environment [9,35]. The objective of the present study is to analyze the longitudinal dynamics of teacher violence and student wellbeing, exploring the potential interplay between these two factors over time in the unique cultural context of rural Sierra Leone where low levels of violence are normative within society. This research aims to provide a more comprehensive understanding of how violence in educational settings affects children’s psychological state. By examining the intertwined development of teacher violence and student wellbeing, we aim to uncover the nuanced relationships between these factors and their implications for individuals’ overall development and adaptation.

## 3. Methods

### Participants and Procedure

The Safe Learning Study was a 5-year project involving approximately 100 schools and 3000 children in northern Sierra Leone. The working assumption behind the Safe Learning Model is that additional support for gender equality, social and emotional learning, and reading will enhance children’s wellbeing and literacy [35]. The aim of the project was to assess the Safe Learning model, an intervention implemented by Concern Worldwide aimed at improving literacy and wellbeing. The intervention had four treatment arms with approximately one-quarter of the cohort of children in each. The intervention had four processes that were layered sequentially across the arms. Support for agriculture and hygiene was in each arm, support for literacy was in three arms, support for social and emotional learning was in two arms, and support for gender equality and community peace was in one arm.

Data were collected in six waves, including two pilot study waves. The data were collected in four waves corresponding with the beginning and end of the school years: November 2018, May 2019, November 2020, and May 2021. All four waves of main data collection were included in this analysis. The participants in the analysis totaled 3170 children, balanced by gender (50.3% boys), with an age range of 5 years to 11 years. The children had varied linguistic backgrounds; at home, 66.8% spoke Temne, 36.4% Krio, 16.6% Kuranko, 17.1% Limba, and the remaining 0.5% Mende. Among the participants, 73% were Muslims, and the remaining 27% were Christian. Regarding parental occupation, 71.6% were employed in the agricultural sector, 11.8% in manual work, and 9.6% in professional or clerical work, and 5.3% were unskilled. Furthermore, 71% of children reported that the person who took care of them at home could not read or write. Participants were given self-report questionnaires. The children were supported if the questions were unclear, and linguistic support was also provided to ensure that everyone could understand the measures.

## 4. Measures

### 4.1. Wellbeing

Wellbeing was measured using the personal subscale of the Child and Adolescent Personal and Social Assessment of Wellbeing (CAPSAW) instrument [36]. The CAPSAW comprises 32 items that assess wellbeing in personal, peer, teacher, and family dimensions. In the current study, the personal dimension subscale of 8 items was used to measure children’s hedonic and eudaimonic wellbeing. Specific items include inquiries about feelings of self-worth, general happiness, life satisfaction, perceived social support, self-perceived abilities, and perceived helpfulness to others (e.g., “Do you think people care about you?” “Can you do things well for yourself?” “Do you think you are helpful to other people?”). Participants were able to respond using a 5-point Likert scale ranging from disagree to agree. The reliability of the instrument was evaluated using Cronbach’s alpha, yielding coefficients ranging from 0.71 to 0.78 across different waves of data collection. This suggests satisfactory internal consistency and reliability of the instrument for assessing psychological wellbeing in the study population. High levels of wellbeing are indicated by high scores on the general mean.

### 4.2. Teacher Violence

The ad hoc tool consists of three questions designed to capture various forms of physical and verbal violence commonly perpetrated by teachers. The forms of violence considered were selected to be representative of those typically encountered in the rural context of Sierra Leone [6,7]. The individual items were developed for the Safe Learning Study to specifically address instances of physical punishment, physical abuse, and verbal abuse inflicted by teachers. Participants are asked to respond to the following inquiries: “Who has ever whipped or caned you? (By a teacher)”; “who has ever beaten you up or physically hurt you? (By a teacher)”; “who has ever called you bad names, teased you, threatened you verbally, or said mean things about you? (By a teacher)”. Participants were able to respond using a 5-point Likert scale ranging from disagree to agree. Cronbach’s alpha ranges from 0.77 to 0.83 across the waves. High levels of teacher violence are indicated by high scores on the general mean.

## 5. Analysis Plan

**Figure 1:**
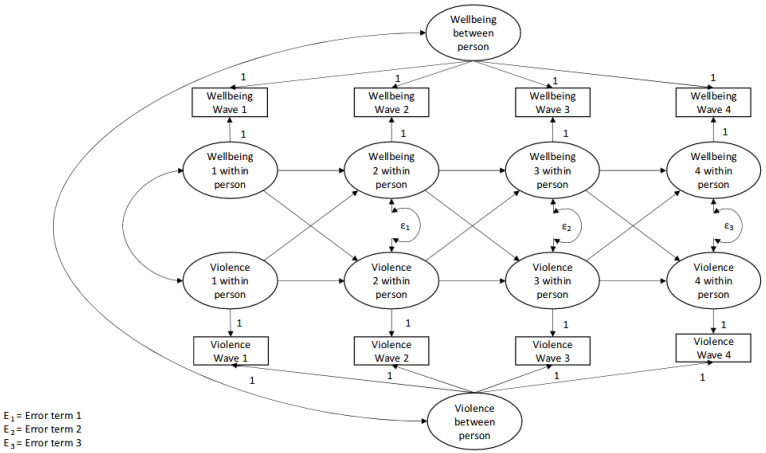
Summary of the model.

We conducted a random intercept cross-lagged panel model (RICLPM) to examine the relationship between violence and mental health across the four waves. The cross-lagged panel model is illustrated in Figure 1, wherein each variable is regressed on its own lagged score as well as the lagged score of the other variable in the model. Cross-lagged panel models offer the advantage of statistically controlling for all other constructs measured at the same time point. Furthermore, the RICLPM parses the relationship between the aspects of the variables that are stable across time (the ‘traits’) across the analysis sample, from the aspects of the variables that vary across individuals across time, meaning that the cross-lagged associations reflect only the relationship of the study variables within individuals rather than across the group. This allows researchers to study group-level traits and individual-level cross-lagged associations in the same model. The analyses were conducted using Mplus version 8.7.

## 6. Results

The model fit the statistics to the data; CFI = 0.98, RMSEA = 0.02. The chi-square test of model fit was significant (X^2^ (9) = 24.90, *p* < 0.01), likely owing in part to the large sample size. The main statistical results of the model are presented in Table 1. We considered *p*-values of <0.05 and below.

At the between-person level, the stable components of student wellbeing and teacher violence across waves 1 to 4 (between-person traits) were positively associated, demonstrating an overall bidirectional relationship between teacher violence and student wellbeing across time. Furthermore, at the within-person level, student wellbeing and teacher violence were positively associated in wave 1. These results indicate a generally positive relationship between wellbeing and violence in the sample.

Regarding autoregressions, there was a lack of stability in student wellbeing and experiences of teacher violence across waves 1 to 3. Between waves 3 and 4, wellbeing and violence experiences became more stable. These results may relate to the timing of the waves. Waves 1 and 2 spanned a school year, and student experiences and teacher behaviors generally change from the start to the end of the year, whereas waves 2 and 3 spanned the COVID-19 school closures, during which environmental disruption could have impacted student wellbeing and teacher behavior. Furthermore, the students were older in subsequent waves, meaning that the patterns of wellbeing and teacher behavior may have stabilized in later waves. It is plausible to suggest that by year’s end, students had become more accustomed to the level of teacher violence to which they were subjected, and this had somewhat of a normalizing effect.

Regarding the cross-lagged pathways, at the within-person level, there was a negative pattern of association between student wellbeing and teacher violence, meaning that having higher wellbeing predicted lower violence and vice versa. The effects were weak and only reached significance for higher student wellbeing for waves 2 and 3, predicting lower teacher violence for waves 3 and 4. These results indicate weak negative relationships between wellbeing and violence for individual students.

Together, the results signal two main things. First, across the cultural context, wellbeing and violence were positively related. Second, having higher wellbeing was a protective factor against experiencing higher teacher violence for individual students.

The model is graphically represented in Figure 1.

## 7. Discussion

The analysis aimed to explore the cross-lagged associations between teacher violence and student wellbeing across four time points in rural Sierra Leone. Time points 1 and 2 were assessed at the beginning and end of the first grade, while time points 3 and 4 were evaluated at the commencement and conclusion of the third grade. Notably, no surveys were conducted during the second grade due to the COVID-19 pandemic. The findings highlight complex and significant relationships between early exposure to violence in the lower grades and wellbeing outcomes in later elementary school years.

### 7.1. Cultural Context and the Impact of Teacher Violence

Overall, the relationship between teacher violence and student wellbeing across the sample and across time was positive, particularly at the start of the study period (wave 1: first grade). This result can be understood within the cultural context, where authoritarianism and strict discipline—even if they involve violence—are often viewed as legitimate methods for promoting success and academic performance. In environments where such practices are normalized, students may internalize these values, coming to associate their sense of wellbeing with conforming to authority and receiving social approval for compliant behavior. In this context, wellbeing may be linked not necessarily to personal fulfillment or emotional health but rather to fulfilling societal expectations and maintaining respect within hierarchies, even if these outcomes are achieved through violent means [27]. This suggests that, for some students, the perceived benefits of adhering to strict disciplinary norms—such as academic success and social acceptance—might outweigh the psychological costs associated with experiencing violence, thereby creating a complex relationship between wellbeing and exposure to teacher violence. However, the longitudinal dynamics between teacher violence and student wellbeing for individual children were found to be weakly negative [35]. This indicates that children with higher levels of wellbeing tended to experience lower levels of teacher violence over time and vice versa. These children likely develop greater resilience and agency in navigating their individual realities, which helps them avoid suffering [37]. Wellbeing may act as a protective factor, allowing children to manage their environments more effectively and potentially mitigate harmful interactions with authority figures [31].

Our finding of a positive association between teacher violence and student wellbeing over time aligns with the literature from West Africa regarding the symbolism of adult violence within the community [7]. In this cultural context, physical punishment and discipline can be interpreted as signs of adults caring for children’s development both at home and in school. Conversely, adults who refrain from physical discipline may be viewed as negligent parents and teachers. However, while children in rural Sierra Leone may come to expect and accept low levels of violence, they are still frightened by it, and it causes them psychological distress [38]. For instance, during a photovoice activity, children in case study communities were asked to take photos of safe and dangerous things in their lives. They distinguished between teachers who were physically violent (unsafe teachers) and those who did not flog or beat them (safe teachers). This aligns with our other findings that greater teacher violence is longitudinally associated with lower student wellbeing. Therefore, another interpretation of the positive relationship between teacher violence and overall student wellbeing in the sample is that other factors might be influencing this relationship, potentially mitigating its effects (e.g., peer support, relationships with parents, and other factors).

### 7.2. Reciprocal Longitudinal Association Between Teacher Violence and WellBeing at the Individual Level

We observed a general pattern of negative longitudinal associations between teacher violence and student wellbeing. While only some of these associations reached statistical significance, the lack of a significant impact of early-grade violence on wellbeing by the end of the first year of elementary school may seem counterintuitive, especially considering the potential severity of violent incidents. This finding suggests that the immediate effects of violence may not be readily apparent in children’s wellbeing outcomes within the short timeframe of the first school year. Moreover, the absence of a significant association between wellbeing at the beginning of first grade and violence by the end of the same grade highlights the complexity of factors influencing children’s experiences with violence. It indicates that individual differences in wellbeing at the start of elementary school do not necessarily lead to experiences of violence later in the academic year. Additionally, the lack of impact of teacher violence at the end of first grade on wellbeing at the beginning of third grade suggests a temporal dissociation between these variables. This finding challenges the assumption that early-grade violence has lasting effects on long-term wellbeing, possibly due to the influence of other factors, such as protective individual or social resources [31]. It indicates that the immediate aftermath of violent incidents may not exert a lasting influence on children’s wellbeing as they transition into subsequent grades. The idea that students may become desensitized to or normalize the violence they experience from teachers over time is a significant consideration. This concept aligns with the theory of desensitization, which suggests that repeated exposure to a stimulus reduces emotional responses to that stimulus. In the context of teacher violence, repeated exposure may lead students to perceive such behavior as more acceptable or less alarming than they initially did. Furthermore, in line with social learning theory [39], individuals learn through observation and modeling the behavior of others. If students witness teacher violence as a common occurrence within their educational environment, they may internalize this behavior as normative.

Another important finding is that lower levels of wellbeing at the end of first grade predict higher levels of violence at the start of third grade, persisting through its conclusion. This longitudinal association suggests a dynamic interplay between wellbeing and violence over time, in which compromised wellbeing serves as a precursor to heightened vulnerability to teacher violence in subsequent school years. At the beginning of a school year, students may be more vulnerable due to being assigned a new teacher, leading to apprehension about the level of violence this new dynamic might entail [40,41]. The lack of significant impact could be attributed to children’s limited awareness of their lived experiences and their nascent ability to attribute emotional and psychological significance to them. They may not fully comprehend the emotional weight of negative interactions, or they may perceive them as normative within their environment. However, the cumulative burden of violence during the mid-grades becomes substantial and enduring, exerting a negative influence on wellbeing. This underscores how wellbeing may depend on the severity of traumatic experiences. Consistent with the existing literature [31], teacher violence may induce a state of asthenia that adversely affects wellbeing, thereby amplifying the impact of risk factors and hindering the search for protective resources to mitigate the negative effects of trauma. From a developmental perspective [42], such traumatic experiences can hinder children’s exploration of essential psychosocial resources for overall wellbeing by disrupting emotional regulation and exacerbating stress.

## 8. Limitations and Future Directions

Despite shedding light on significant facets pertinent to children in Sierra Leone, this study is subject to certain limitations. The utilization of self-report questionnaires, while translated, may introduce biases or magnify perceptions. Another concomitant limitation concerns the wide age range, which may pose challenges in terms of measurement equivalence, particularly regarding the possible cognitive limits of young children. Future investigations, for instance, could employ observational methodologies to delve deeper into the understanding of welfare dynamics and disciplinary practices. Future studies may also explore additional protective factors, particularly in shaping preventive interventions. Another limitation is the use of a questionnaire to measure teacher-perpetrated violence, as it may overlook other relevant and specific details about teacher punishment. Including more detailed information on the nature of the punishment could provide additional insights to better understand the puzzling findings in the Sierra Leone study [43,44]. However, while the information is important for understanding the dynamics of wellbeing and violence, one limitation is that we did not control for the effects of the interventions conducted. Future studies in Sierra Leone could specifically focus on the effects of interventions aimed at combating violence. This study also underscores the imperative of promoting public and educational policies that counteract violence, preventing children from internalizing the detrimental effects of intergenerational violence. Indeed, such effects, by impairing wellbeing, may manifest in subsequent forms of violence or compromise other aspects of social relationships.

## 9. Conclusions

This study underscores the importance of monitoring and improving teacher–student relationships to prevent mental health issues and promote a positive school climate in Sierra Leone [45]. It highlights the urgent need to implement public and educational policies aimed at reducing violence, particularly in Sierra Leone’s schools, by fostering a culture of nonviolence. Promoting positive mental health, creating supportive school environments, and equipping teachers with training in nonviolent discipline techniques are key strategies for achieving this goal. Preventing teacher violence must be integrated into broader initiatives that focus on child wellbeing and the protection of children’s rights. A comprehensive, multifaceted approach is essential for building safer, more nurturing educational environments where students can flourish both academically and emotionally. This approach should include the use of evidence-based programs that address various dimensions of student wellbeing, safety, and the promotion of nonviolent values. By focusing on prevention and the adoption of positive educational practices, schools can contribute to the long-term development of a nonviolent culture, ensuring that students grow in environments that respect their dignity and foster their potential.

## Figures and Tables

**Table 1:** Model results.

Model Pathways	*b*	*SE*	*t*	*p*
Between-person waves 1–4 covariance	1.19	0.45	2.65	0.008
Within-person wave 1 covariance	0.15	0.04	3.72	0.000
Residuals wave 2 covariance	−0.08	0.03	−2.23	0.026
Residuals wave 3 covariance	0.04	0.03	1.23	0.218
Residuals wave 4 covariance	−0.06	0.05	−1.33	0.184
**Autoregressions**				
Wellbeing wave 2 on wellbeing wave 1	0.06	0.03	1.98	0.048
Wellbeing wave 3 on wellbeing wave 2	0.00	0.04	−0.12	0.903
Wellbeing wave 4 on wellbeing wave 3	0.16	0.03	4.66	0.000
Violence wave 2 on violence wave 1	0.03	0.04	0.69	0.493
Violence wave 3 on violence wave 2	−0.02	0.04	−0.49	0.624
Violence wace 4 on violence wave 3	0.07	0.03	2.21	0.027
**Cross-lagged pathways**				
Wellbeing wave 2 on violence wave 1	−0.01	0.03	−0.47	0.640
Wellbeing wave 3 on violence wave 2	−0.02	0.03	−0.52	0.606
Wellbeing wave 4 on violence wave 3	−0.05	0.03	−1.43	0.152
Violence wave 2 on wellbeing wave 1	−0.04	0.03	−1.34	0.181
Violence wave 3 on wellbeing wave 2	−0.07	0.03	−2.21	0.027
Violence wave 4 on wellbeing wave 3	−0.09	0.04	−2.49	0.013

## Data Availability

Data can be requested by the author(s).
